# Impact of anastomotic technique and norepinephrine on microcirculation in colorectal surgery: findings from a porcine model using laser speckle contrast imaging

**DOI:** 10.1007/s10151-025-03195-3

**Published:** 2025-07-19

**Authors:** R. Paramasivam, R. Ambrus, N. M. Kristensen, M. Stavsetra, C. Jaensch, M. W. Ørntoft, A. H. Madsen

**Affiliations:** 1 Department of Surgery, Godstrup Hospital, Herning, Denmark; 2 Department of Clinical Medicine, Aarhus University, Aarhus, Denmark; 3 NIDO | Centre for Research and Education, Godstrup Hospital, Herning, Denmark

**Keywords:** Colorectal cancer, Intestinal anastomoses, Microcirculation, Porcine model, Surgery

## Abstract

**Background:**

Proper intestinal anastomosis healing in colorectal surgery relies on optimal microcirculation, with surgeons choosing between the hand-sewn and stapled techniques. However, the impact of these methods on the microcirculation remains unclear. This study used laser speckle contrast imaging (LSCI) to objectively assess the impact of hand-sewn and stapled techniques on microcirculation in a porcine model during open surgery and examined microcirculatory changes during hypotension and norepinephrine (NE) correction.

**Methods:**

Ten healthy female pigs underwent midline laparotomy, with one hand-sewn and one stapled anastomosis in both the small intestine and colon. LSCI measurements were obtained before creation (baseline), immediately after anastomosis (*T*_0_), after 1 h of rest (*T*_60_), during induced hypotension, and after NE infusion. Measurements were performed directly on the anastomosis, adjacent tissue, and an untouched area of the intestine.

**Results:**

At *T*_0_, microcirculation significantly decreased across all anastomosis types, with hand-sewn anastomoses experiencing a greater decline than stapled anastomoses. An improvement was noted at *T*_60_ for all anastomoses. Hypotension worsened microcirculation in all anastomosis types, and NE infusion did not improve microcirculation despite increased and stabilized mean arterial pressure (MAP).

**Conclusions:**

Stapled anastomoses initially exhibited superior microcirculation compared with hand-sewn anastomoses, but the disparity disappeared after 1 h. Hypotension significantly impairs simple anastomotic microcirculation. Moreover, while NE is effective in stabilizing the general blood pressure, it contributed to further diminishment in intestinal microcirculation, especially around anastomoses. Thus, the use of NE postoperatively may be considered a risk factor for anastomotic leakage.

**Supplementary Information:**

The online version contains supplementary material available at 10.1007/s10151-025-03195-3.

## Introduction

Anastomotic leakage (AL) is one of the most severe complications of colorectal surgery and is directly linked to increased mortality and morbidity [1]. AL significantly reduces the 5-year survival rate and negatively affects the quality of life of patients [2–4]. Despite advancements in surgical techniques, the incidence of AL remains notably high [5].

Various anastomotic techniques, including hand-sewn and stapled approaches, have been developed to minimize the risk of AL [6]. Hand-sewn anastomoses involve manual suturing for a tight seal, while stapled anastomoses use surgical staplers for a quicker and more uniform connection. Numerous surveys and studies have been conducted to determine whether one type of anastomotic technique carries a lower risk of AL than the other [7], but the jury is still out [7–9]. Adequate blood supply is crucial for anastomotic healing, making optimal perfusion a key factor in preventing AL [10–12]. However, the effects of different anastomotic techniques on microcirculation in various clinical scenarios have not yet been described.

Inotropic agents, particularly norepinephrine (NE), are crucial in managing hemodynamic stability during and after colorectal surgery. NE, a potent vasopressor, is commonly used to maintain blood pressure and perfusion [13]. However, its effects on microcirculation, especially in intestinal tissue, are complex and concerning; NE can induce vasoconstriction, potentially compromising microcirculation and leading to cellular ischemia [14, 15]. This is especially critical for intestinal anastomoses, where optimal microcirculation is essential for healing [16, 17]. Understanding the effect of NE on intestinal microcirculation is vital for optimizing surgical outcomes and patient recovery after intestinal anastomosis.

Currently, the assessment of perfusion at the anastomotic site is entirely subjective, relying on indicators such as palpable arterial pulsation, intestinal color, and bleeding from the surgical edges. These assessments lack objectivity, quantifiability, and predictive accuracy [18] and do not specifically evaluate the microcirculation. Therefore, tools to support perioperative evaluation of microcirculation are emerging. Among these, laser speckle contrast imaging (LSCI) is a promising and validated technique [19–21] that enables real-time, dye-free visualization of microvascular blood flow. It works by analyzing the dynamic speckle patterns formed when laser light is scattered by moving red blood cells within the tissue, providing a detailed map of tissue perfusion. LSCI has been validated in both preclinical and clinical studies across various surgical and medical disciplines [22]. Furthermore, LSCI is currently being introduced into clinical practice in accordance with the IDEAL framework [23–25] for surgical innovation (stage 2a/2b), where early feasibility and exploration studies are ongoing to define its role in intraoperative decision-making. This includes its application in colorectal surgery to assess anastomotic perfusion and potentially reduce the risk of anastomotic leakage through objective perfusion assessment [19, 26, 27]. Consequently, continued preclinical investigation in line with IDEAL stage 0 remains important to further explore the full applicability of LSCI, including its reliability across repeated measurements and technical robustness in various surgical situations.

To assess the intricate relationship between microcirculation and intestinal anastomoses in different clinical scenarios using LSCI, in line with the IDEAL framework, we designed a porcine model with the following aims:

1. To investigate and compare how microcirculation is influenced by hand-sewn versus stapled intestinal anastomoses.
2. To examine how microcirculation in hand-sewn and stapled anastomoses is affected by hypovolemic hypotension and to explore how NE administration may affect the anastomotic microcirculation.
3. To examine LSCI as a tool for perioperative evaluation of anastomotic microcirculation.

## Materials and methods

The impact of hand-sewn versus stapled anastomoses on microcirculation was assessed in ten pigs using an experimental setup, IDEAL stage 0. Microcirculation was measured using LSCI.

### Anesthesia

Anesthetic administration in the porcine model has been previously described [21]. In brief, ten female pigs (Danish Landrace/Yorkshire/Duroc), averaging 41.75 ± 1.58 kg and aged 14–16 weeks, were sedated, and anesthesia was induced via peripheral intravenous catheters and maintained with propofol and fentanyl. The pigs were intubated and mechanically ventilated and received Lactated Ringer’s solution at 5 ml/kg/h. Urinary and 8F arterial catheters were used to monitor the circulation. Arterial blood gas analyses were performed to monitor saturation and overall condition.

### Laser speckle contrast imaging

LSCI was used to assess the microcirculation at a wavelength of 785 nm (MoorFLPI-2, Moor Instruments, Axminster, UK). This technique evaluates the microcirculation at a depth of approximately 1 mm. The LSCI system was fixed at a height of 25 cm, covering at least 5 cm of the intestine on each side of the region of interest (ROI). Each measurement was recorded for 30 s, with a sampling rate of 25 frames per second [28]. Perfusion was quantified in two ways: (1) as a color-coded picture where red color indicates high microcirculation and blue color indicates poor microcirculation, and (2) as laser speckle perfusion units (LSPU), which are arbitrary units derived from speckle contrast analysis by a computer algorithm; higher LSPU values indicate greater microcirculatory perfusion, while lower values reflect reduced blood flow.

### Surgical procedure

**Fig. 1:**
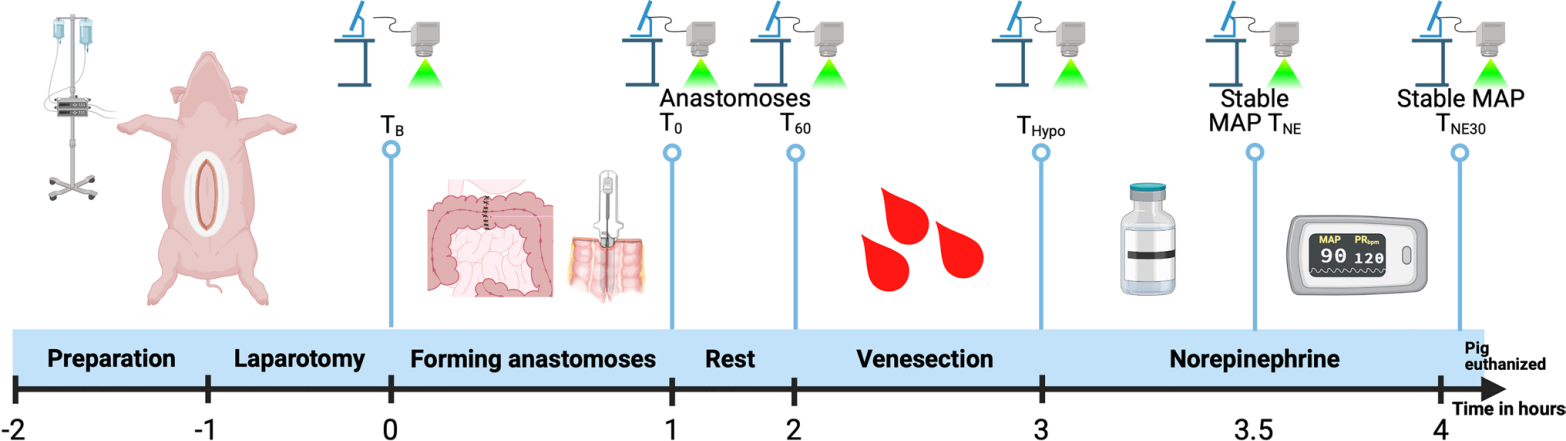
Porcine model timeline. Overview of the experiment detailing all steps and time points for LSCI measurements. *T*_B_ = baseline; *T*_0_ = immediately after anastomosis; *T*_60_ = 1 h with rest; *T*_Hypo_ = hypotension, MAP 50–60 mmHg; *T*_NE_ = restored MAP 85–100 mmHg with NE infusion; *T*_NE30_ = after 30 min of MAP 85–100 mmHg with continuous NE. MAP, mean arterial pressure; NE, norepinephrine

Following the induction of general anesthesia, midline laparotomy was performed to expose the colon and small intestines. For each pig, a single-layered, seromuscular hand-sewn end-to-end anastomosis [29] and one stapled anti-mesenteric side-to-side anastomosis [30] were performed on both the small intestine and the colon, resulting in four anastomoses in total. All anastomoses were performed by two experienced surgeons to ensure consistency. LSCI measurements were conducted on all four anastomoses, as well as on the untouched small intestine and colon, in a standardized manner (Fig. 1). Baseline measurements (*T*_B_) were recorded before anastomosis formation. *T*_0_ measurements were recorded immediately after anastomosis. The pig was then allowed to rest for an hour without any surgical intervention, and the *T*_60_ was measured. In the next step of the procedure, hypotension was induced by rapid bleeding through venesection from one of the arterial catheters until the desired mean arterial pressure (MAP) of 50–60 mmHg was achieved. Then, *T*_Hypo_ was measured. After LSCI measurements, NE infusion was initiated to achieve a MAP of 85–100 mmHg, and *T*_NE_ was measured. After the MAP had been stable for 30 min during continuous NE infusion, *T*_NE30_ was measured, and the pig was euthanized.

### Computational and statistical analysis

**Fig. 2:**
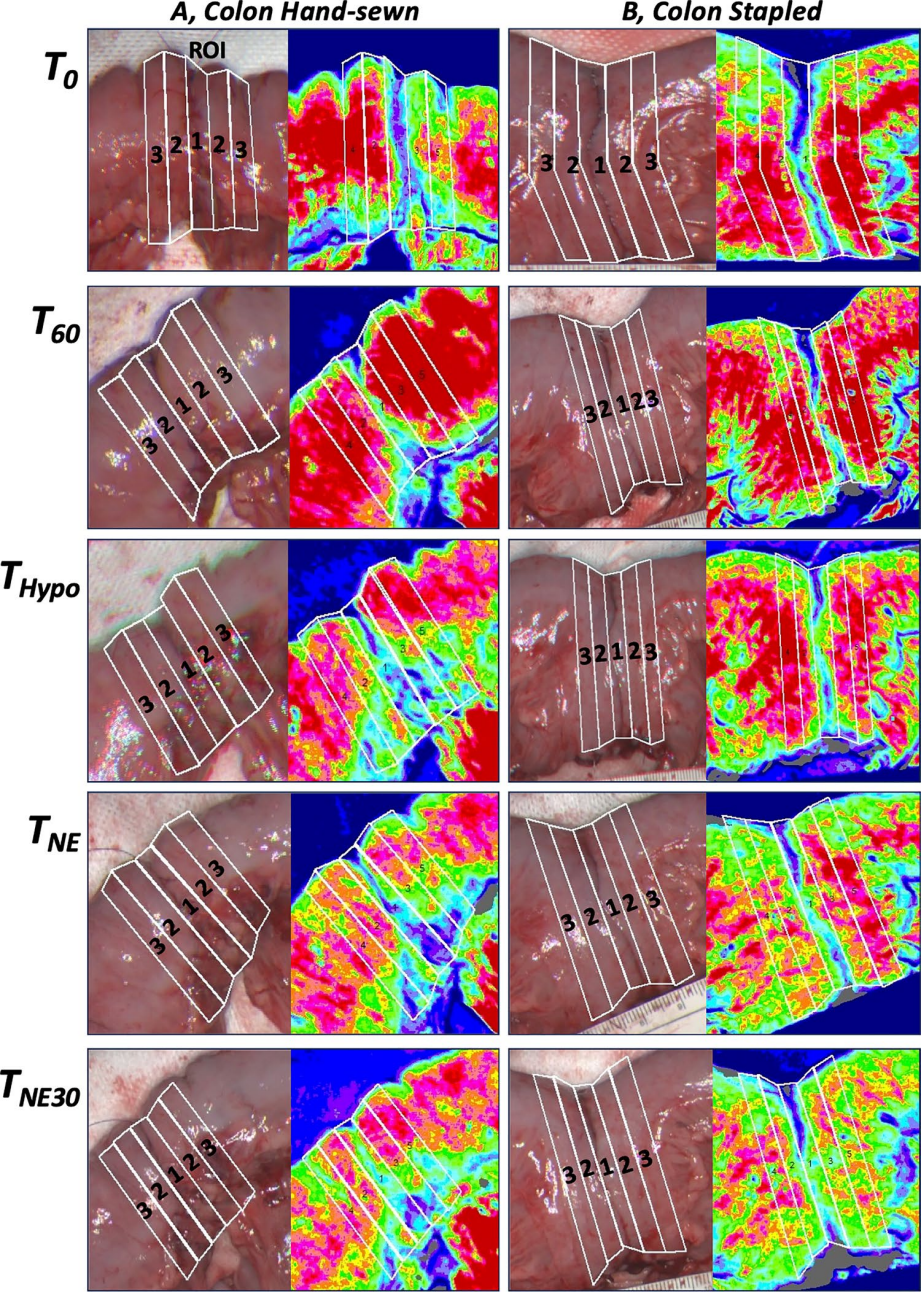
Examples of LSCI measurements of colonic anastomoses: (A) hand-sewn; (B) stapled. The placement of regions of interest (ROIs) is indicated on all white light images using bold markings: ROIs were placed adjacent to each other, with ROI 1 centered on the anastomosis, ROI 2 ± 5 mm on either side, and ROI 3 ± 10 mm on either side of the anastomosis. A color scale ranging from red to blue indicates good to poor perfusion, respectively. *T*_B_ = baseline; *T*_0_ = immediately after anastomosis; *T*_60_ = 1 h with rest; *T*_Hypo_ = hypotension, MAP 50–60 mmHg; *T*_NE_ = restored MAP 85–100 mmHg with NE infusion; *T*_NE30_ = after 30 min of MAP 85–100 mmHg with continuous NE. MAP, mean arterial pressure; NE, norepinephrine

Four ROIs were defined for the analysis (Fig. 2): ROI 1 was placed directly on the anastomosis, ROI 2 was positioned 5 mm from the anastomosis on either side, ROI 3 was located 10 mm from the anastomosis on either side, and the final ROI was placed on an untouched section of the intestine. Each ROI was 5 mm wide and covered the entire diameter of the intestine, and ROI 1–3 were adjacent to each other. Median values, measured in LSPU, were calculated for each ROI and tested for normal distribution. The results were analyzed using linear mixed models for repeated measurements within pigs and are presented as % difference in LSPU at a given time, compared with the LSPU baseline established across all ten animals in the study. Statistical significance was set at *P* < 0.05. Statistical analysis was performed using STATA version 18 (StataCorp LLC, College Station, TX, USA). Flux data were analyzed using MoorFLPI2 Research software version 2.x (Moor Instruments, Axminster, UK).

### Ethics

The trial was conducted under the supervision of veterinary personnel at the Department of Animal Science, Aarhus University, AU Foulum, and the Principles of Laboratory Animal Care [31] were followed. The trial was approved by the Danish Animal Experiments Inspectorate (no. 2020-15-0201-00695).

## Results

**Fig. 3:**
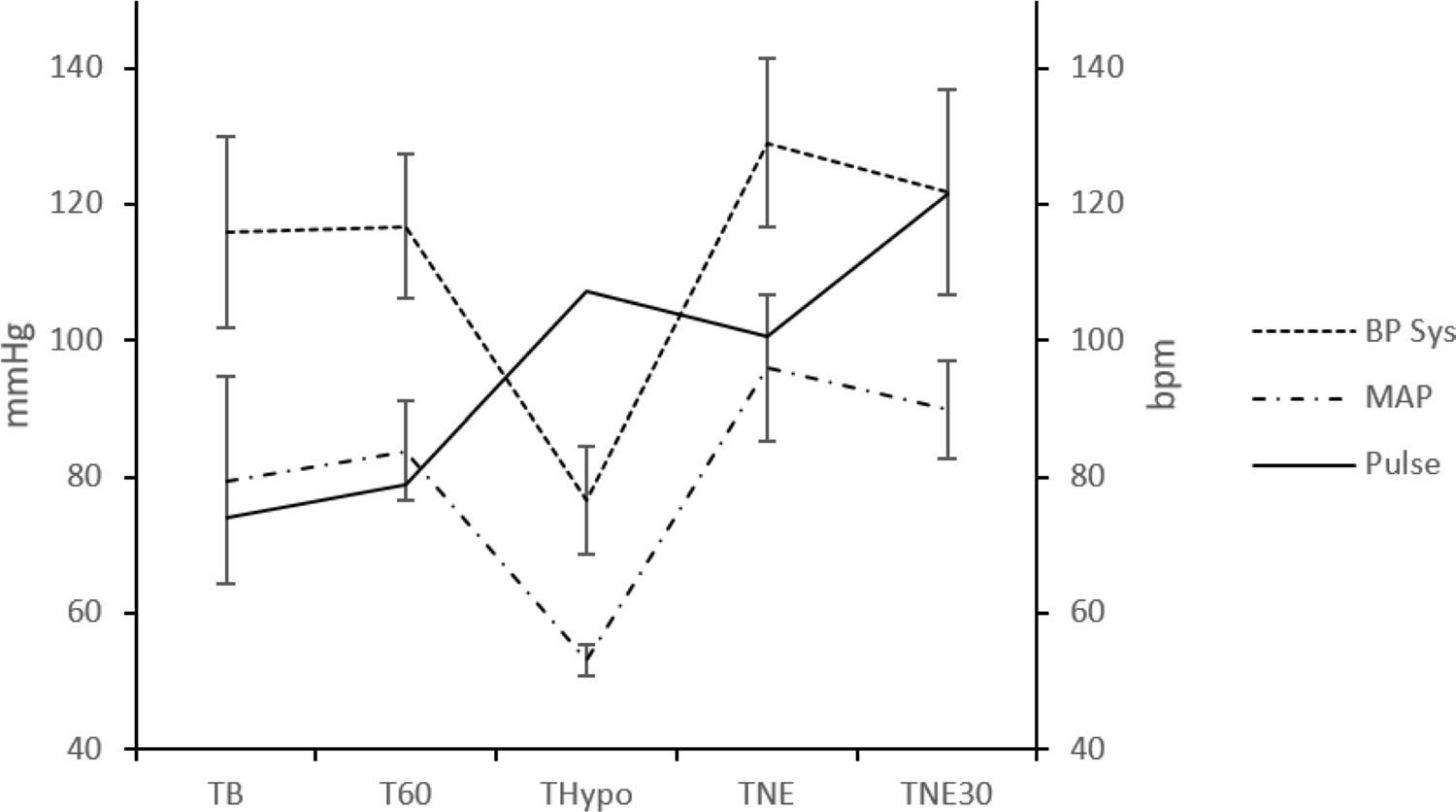
Hemodynamic variables in the porcine model. The graph shows the mean value ± standard deviation (SD) (whiskers). *X*-axis represents time points: *T*_B_ = baseline; *T*_60_ = 1 h with rest; *T*_Hypo_ = hypotension, MAP 50–60 mmHg; *T*_NE_ = restored MAP 85–100 mmHg with NE infusion; *T*_NE30_ = after 30 min of MAP 85–100 mmHg with continuous NE. The *y*-axis represents values in mmHg or beats per minute (bpm). BP Sys, systolic blood pressure; MAP, mean arterial pressure; NE, norepinephrine

**Fig. 4:**
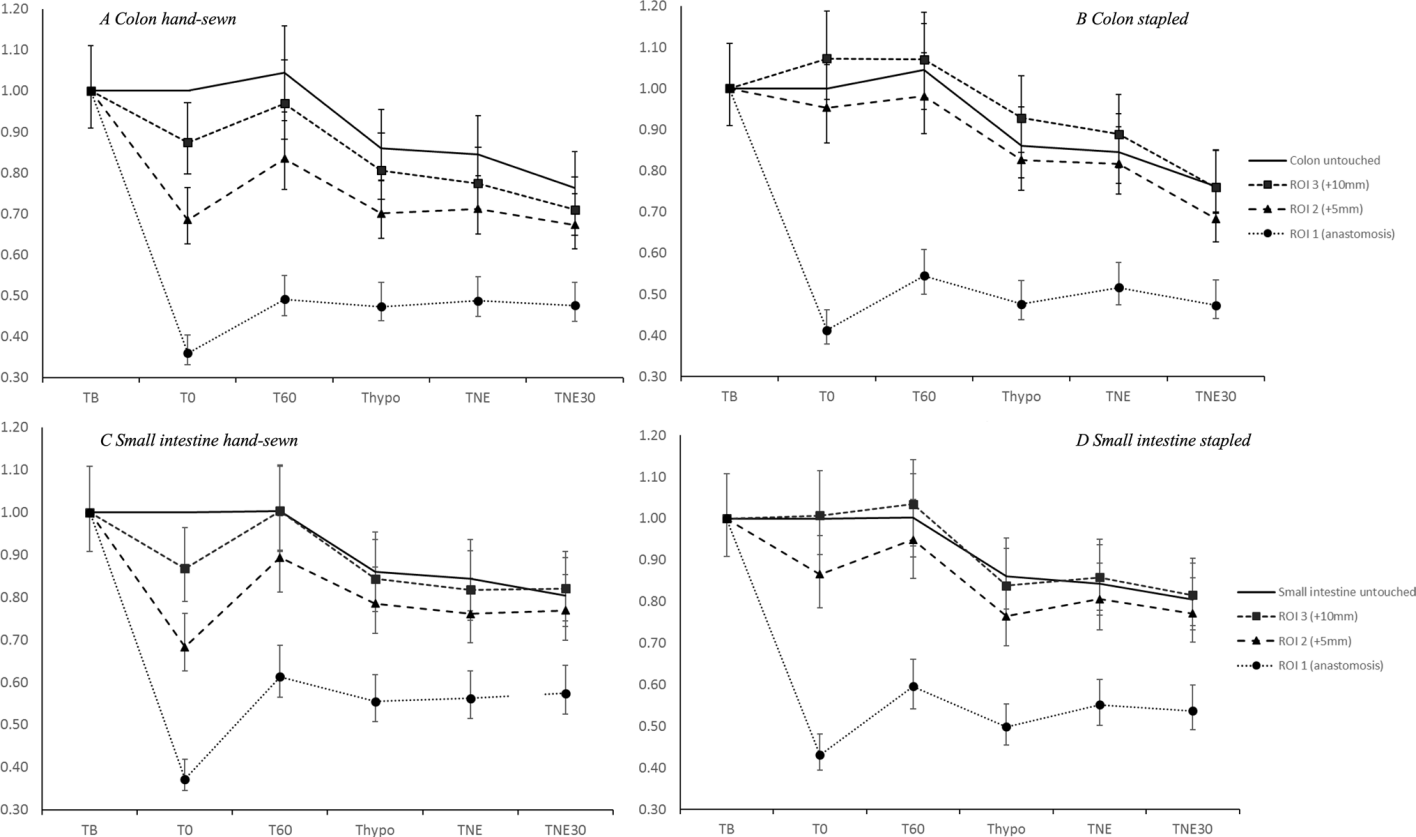
Graphical representation of the relative change in microcirculation from baseline (whiskers represent 95% confidence interval (CI)). The *x*-axis represents time points: time points: *T*_B_ = baseline; *T*_0_ = measurements taken immediately after the anastomosis was created; *T*_60_ = measurements taken after a 1-h rest period; *T*_Hypo_ = hypotension; *T*_NE_ = norepinephrine with stable MAP; *T*_NE30_ = infusion of norepinephrine for 30 min. The *y*-axis represents the relative change. NE, norepinephrine; CI, confidence interval; MAP, mean arterial pressure

Ten pigs underwent the surgical procedures with no complications or adverse events. To induce targeted hemodynamic changes, an average of 752 ml blood was aspirated (min:max, 350:1400 ml) during the rapid bleeding phase. The hemodynamic data are shown in Fig. 3. LSCI measurements were performed at all time points in all pigs. Examples of LSCI images of the microcirculation around the anastomoses are shown in Fig. 2 (colon) and Supplementary Fig. 1 (small intestine). LSPU values are presented as relative changes compared with baseline in Fig. 4 and as absolute LSPU values in Supplementary Table 1.

### Microcirculation in untouched intestine during surgery

In the untouched colon and small intestine, there were no significant changes in the microcirculation from *T*_B_ to *T*_60_ after 1 h of rest. However, during hypovolemic hypotension, the microcirculation decreased significantly by 14% in both the colon and small intestine. After 30 min of NE infusion, the microcirculation continued to decline, dropping by 24% in the colon and 20% in the small intestine.

### Microcirculatory changes in anastomosis at baseline and after 1 h’s rest compared with untouched tissue

In summary, in all anastomoses, at both *T*_0_ and *T*_60_, the microcirculation decreased most at the resection line and improved as the distance to the resection line increased above 7.5 mm. Furthermore, hand-sewn anastomoses were affected more than stapled anastomoses, and colonic microcirculation decreased more than microcirculation in the small intestine. After 1 h of rest, the microcirculation improved across all anastomoses and ROIs, although hand-sewn anastomoses in the colon still exhibited lower microcirculation than stapled anastomoses. No significant changes were observed in ROI 3 for any of the anastomosis types.

### Microcirculatory changes during hypotension, NE infusion, and staple MAP compared with untouched tissue

In summary, in all anastomoses at *T*_Hypo_, the microcirculation decreased. Normalizing blood pressure with NE initially led to stagnation of microcirculation without improvement. With continuous NE administration, there was a further decline in the microcirculation across all ROIs. The colon was more affected than the small intestine, and eventually, both hand-sewn and stapled anastomoses were equally affected in both the colon and the small intestine, with no significant difference.

### IDEAL stage 0

In line with the IDEAL stage 0 framework, the present study evaluated LSCI from a clinical perspective as a noninvasive and low-risk technology. The perfusion patterns observed using LSCI—following anastomosis creation, venesection, and norepinephrine administration—closely mirrored what one would expect on the basis of clinical experience, thereby supporting the physiological validity of the measurements. The method proved to be user-friendly, allowed for repeated assessments across a prolonged surgical procedure, and enabled intuitive interpretation of results. Given the negligible risk and technical ease of use, LSCI can be categorized as a low-risk investigative tool in this early stage research setting.

## Discussion

In this study, we compared the effects of hand-sewn versus stapled intestinal anastomoses on microcirculation using LSCI. Our findings indicate an initial significant difference in microcirculation in the tissue adjacent to the anastomosis between the hand-sewn and stapled anastomoses. However, over the course of an hour, we observed substantial improvement, although a small significant difference persisted. Hypovolemic hypotension compromised microcirculation around all anastomoses. Notably, while NE administration improved blood pressure, it also had a significant negative impact on microcirculation around the anastomoses in general. This effect was also clearly observed in the untouched intestine, confirming the systemic impact of both hypotension and norepinephrine on intestinal microcirculation.

### Differences in hand-sewn versus stapled anastomoses

Our study found that both hand-sewn and stapled anastomoses exhibited a substantial drop (approximately 60%) in microcirculation at the resection line (ROI 1) and adjacent to the anastomosis (ROI 2), which improved substantially after 1 h’s rest. We demonstrated a small significant difference between the two types of anastomoses in the colon. However, clinical studies have not shown a significant difference in leakage rates between hand-sewn and stapled anastomoses [6–8].

This discrepancy may be due to the difficulty in clinically detecting small differences in the microcirculation without very large cohorts. In addition, the impact of other factors on anastomotic healing, such as sepsis and comorbidities, likely outweighed the minor differences in microcirculation between the two techniques. Moreover, caution should be exercised in interpreting this small significant difference observed in this study, since confidence intervals overlapped and the marked improvement during 1 h’s rest for the hand-sewn anastomosis may not have reached its plateau yet. If measurements were taken after additional rest, microcirculatory differences between anastomosis types might be diminished or even erased. This finding suggests that both techniques, from a microcirculatory standpoint, have equal healing potential.

The finding of an initially slightly more pronounced decline in the microcirculation of hand-sewn anastomoses, regardless of intestinal type, is likely due to the more extensive tissue manipulation involved in hand-sewn anastomoses where the stitches “bite” more tissue compared with stapling. This result aligns with a study from 2022, in which the authors compared 21 stapled and 25 hand-sewn anastomoses, all in the small intestine [32], and the microcirculation was assessed immediately after the anastomoses were created using hyperspectral imaging. Similar to our data, they found reduced microcirculation in hand-sewn anastomoses but identified no clinical correlation with AL [32]. However, in our study, the microcirculation was also measured after 1 h of rest without further surgical intervention, which showed a marked improvement in microcirculation. This could explain why microcirculatory measures at *T*_0_ by Wagner et al. have little predictive value in AL; subsequently, we hypothesized that measurement of microcirculation after 1 h could be a better predictor of AL.

### Differences in the small intestine versus colon

Our results showed that for the small intestine, the microcirculatory difference between hand-sewn and stapled anastomoses equalized after 1 h and that the small intestinal microcirculation in general was less affected by hypotension and NE infusion than colonic microcirculation. The greater resilience and ability to restore adequate blood flow after surgery in the small intestine, regardless of the anastomosis type, could explain what existing literature and clinical experience have empirically reported: the leakage rate for entero–entero anastomoses is generally very low [33].

In contrast, a persistent microcirculatory decrease was seen for colonic hand-sewn anastomosis, even after 1 h of rest. This can be attributed to several factors. The colonic blood supply is more segmented and less redundant than that in the small intestine, making it more vulnerable to ischemia [34]. In addition, the thicker and less vascularized tissue of the colon could account for the slower recovery of the microcirculation observed in our study.

### NE impact on microcirculation

NE increases blood pressure through vasoconstriction, which is crucial during hypotensive states for maintaining vital organ perfusion. However, vasoconstriction does not uniformly improve microcirculation, particularly in the intestines [13–15]. Our study found that when hypovolemic hypotension was reversed with NE to achieve normotension, intestinal microcirculation declined across all ROIs, indicating that NE-stabilized MAP did not improve intestinal microcirculation. Continuous NE administration caused an immediate and sustained decline in microcirculation, suggesting that while NE restores systemic blood pressure, it impairs the intestinal blood supply.

Previous studies have linked vasopressor use, such as NE, with an increased risk of AL [17, 35, 36]. Our findings support this physiologically, showing that NE affects microcirculation around anastomoses within the first 5 mm (ROI 2) more than in healthy tissues, potentially due to the heightened vulnerability of healing tissues to vasoconstriction. Moreover, previous research has established that the lower limit of acceptable perfusion is approximately a 25% reduction [37]. In our porcine model, 30 min of NE administration reduced microcirculation to this critical threshold or lower. While systemic blood pressure normalizes, the intestinal blood supply does not necessarily follow, leading to diminished microcirculation after MAP correction with NE.

This reduced microcirculatory flow following NE-induced MAP correction was significant. Thus, postoperative use of NE could be a direct risk factor for AL by reducing the microcirculation around anastomoses. However, NE is often used in critically ill patients, and is thus associated with patients in compromised physiological state, which is also associated with increased risk of AL. Therefore, it will be difficult to prospectively examine the isolated microcirculatory effect of NE on anastomoses in a clinical cohort. Still, the potential risk of impaired microcirculation and poor healing at the anastomosis site should be carefully weighed against the need for increased MAP pressure when using NE as the vasopressor of choice.

### Clinical utility and future potential of LSCI

Our findings suggest that LSCI is a feasible and valuable adjunct for intraoperative assessment of intestinal microcirculation in colorectal surgery. From an IDEAL perspective, this technique appears well suited for perioperative application, offering real-time feedback without interfering with the surgical field. Its ability to detect microcirculatory changes in response to surgical and hemodynamic interventions may add new dimensions to surgical decision-making. Therefore, LSCI holds potential as a clinically useful tool to enhance intraoperative judgment, particularly in evaluating anastomotic perfusion.

### Limitations

When interpreting LSCI data, speckle patterns are converted into LSPU values, which are arbitrary units that can vary significantly. In this study, we analyzed the mean progression of LSPU values over time compared with the mean baseline values because assessment of relative changes seems to provide the most effective interpretation [38].

Our choice of ROI placement was made to allow direct assessment of the microcirculation at the anastomosis and its surrounding areas. Visual examination of LSCI video sequences at ROI 1 shows how the blood supply surrounds the sutures or staples, allowing for a reliable assessment of adjacent tissue. However, artifacts from sutures or staples contribute to the significant decline in microcirculation at ROI 1, resulting in very low LSPU values (indicated by the blue color in Fig. 2).

The present study design had several limitations when extrapolating the results to a clinical context. We utilized a porcine model because conducting LCSI measurements under comparable standardized conditions in humans is unfeasible. Consequently, these findings may not be directly applicable to humans, although porcine models are widely accepted as the best means of translating experimental results to human intestinal physiology [39]. Another constraint is the relatively small number of porcine experiments. Small studies can lead to type II errors, where the existing differences may be too subtle to reach statistical significance. Nevertheless, we achieved our objectives and consistently demonstrated the intended effects while taking ethical considerations regarding animal model research into account [40].

## Conclusions

Initially, stapled anastomoses demonstrated better blood supply than hand-sewn anastomoses, but this difference equalized within 60 min. Hypotension caused by bleeding leads to decreased microcirculation, and while NE effectively stabilizes MAP pressure, it aggravates the reduction in intestinal microcirculation around the anastomoses. LSCI was effective and harmless for perioperative assessment of microcirculation in anastomoses.

Further studies are needed to determine whether the NE-induced reduction in microcirculation, and consequently the postoperative use of NE in colorectal surgery, may be considered a risk factor for AL.

## Supplementary Information

Below is the link to the electronic supplementary material.

Supplementary file1 (DOCX 11031 KB)

## Data Availability

All data generated or analyzed during this study are included in this article and its supplementary material files. Further inquiries can be directed to the corresponding authors.
