# Supplementary material for: Impact of anastomotic technique and norepinephrine on microcirculation in colorectal surgery: findings from a porcine model using laser speckle contrast imaging
Source: Tech Coloproctol. 2025 Jul 19;29(1):144. doi: 10.1007/s10151-025-03195-3 (PMC12276112; doi:10.1007/s10151-025-03195-3)
Supplement: Supplementary file 1 — Supplementary file1 (DOCX 11031 KB) [file 10151_2025_3195_MOESM1_ESM.docx]

|  | Baseline | T_0_ | *p-value* | T_60_ | *p-value* | T_Hypo_ | *p-value* | T_NE_ | *p-value* | T_NE30_ | *p-value* |
| --- | --- | --- | --- | --- | --- | --- | --- | --- | --- | --- | --- |
| Colon untouched | 1007 (909-1106) (ref.) |  |  | 1052 (963-1141) | 0.380 | 866 (776-955) | 0.028* | 851 (758-944) | 0.027* | 768 (641-895) | 0.019* |
| Microcirculation in colon hand-sewn |  |  |  |  |  |  |  |  |  |  |  |
| Anastomosis |  | 363 (288-439) | 0.000* | 495 (400-591) | 0.000* | 477 (355-598) | 0.000* | 491 (388-594) | 0.000* | 480 (389-572) | 0.000* |
| ±5mm |  | 690 (591-789) | 0.002* | 841 (757-925) | 0.013* | 706 (609-803) | 0.001* | 717 (617-817) | 0.001* | 678 (589-768) | 0.001* |
| ±10mm |  | 880 (773-987) | 0.035* | 976 (881-1072) | 0.479 | 812 (716-908) | 0.003* | 780 (681-879) | 0.001* | 715 (620-811) | 0.001* |
|  |  |  |  |  |  |  |  |  |  |  |  |
| Microcirculation in colon staplet |  |  |  |  |  |  |  |  |  |  |  |
| Anastomosis |  | 416 (337-495) | 0.000* | 549 (448-650) | 0.000* | 480 (389-571) | 0.000* | 520 (421-619) | 0.000* | 477 (330-624) | 0.000* |
| ±5mm |  | 960 (870-1050) | 0.441 | 988 (911-1065) | 0.772 | 832 (742-922) | 0.016* | 823 (746-900) | 0.003* | 688 (560-815) | 0.004* |
| ±10mm |  | 1080 (998-1162) | 0.178 | 1078 (1000-1157) | 0.282 | 935 (835-1035) | 0.297 | 895 (816-974) | 0.035* | 765 (634-896) | 0.020* |
|  |  |  |  |  |  |  |  |  |  |  |  |
| Small intestine untouched | 1573 (1444-1703) (ref.) |  |  | 1577 (1490-1664) | 0.964 | 1353 (1237-1469) | 0.018* | 1327 (1197-1456) | 0.025* | 1266 (1146-1385) | 0.027* |
| Small intestine Hand-sewn |  |  |  |  |  |  |  |  |  |  |  |
| Anastomosis |  | 585 (419-751) | 0.000* | 965 (766-1164) | 0.000* | 873 (746-1000) | 0.000* | 884 (740-1028) | 0.000* | 903 (779-1027) | 0.000* |
| ±5mm |  | 1075 (901-1250) | 0.001* | 1406 (1274-1539) | 0.152 | 1236 (1119-1354) | 0.009* | 1197 (1068-1326) | 0.002* | 1210 (1104-1317) | 0.014* |
| ±10mm |  | 1365 (1209-1522) | 0.041* | 1579 (1456-1702) | 0.956 | 1327 (1201-1453) | 0.034* | 1287 (1142-1432) | 0.020* | 1291 (1200-1382) | 0.028* |
|  |  |  |  |  |  |  |  |  |  |  |  |
| Small intestine Stapled |  |  |  |  |  |  |  |  |  |  |  |
| Anastomosis |  | 678 (564-792) | 0.000* | 938 (856-1020) | 0.000* | 785 (706-863) | 0.000* | 868 (773-962) | 0.000* | 845 (722-969) | 0.000* |
| ±5mm |  | 1362 (1263-1462) | 0.001* | 1492 (1452-1533) | 0.355 | 1203 (1123-1282) | 0.003* | 1268 (1172-1364) | 0.006* | 1213 (1083-1342) | 0.028* |
| ±10mm |  | 1584 (1481-1688) | 0.797 | 1627 (1576-1678) | 0.487 | 1319 (1225-1414) | 0.017* | 1350 (1246-1454) | 0.021* | 1283 (1164-1402) | 0.049* |

**Suppl. Table 1: Microcirculatory changes in colonic and small intestine anastomoses in porcine model**LSCI measurements were presented as the mean laser speckle perfusion unit (LSPU) value (95% CI).
Anastomosis: ROI 1, ± 5 mm; ROI 2, ± 10 mm; ROI 3, untouched intestine. Time points: T_0_ measurements taken immediately after anastomosis creation; T_60_ measurements taken after a 1-hour rest period; T_Hypo_, hypotension; T_NE_, norepinephrine stable MAP; T_NE30_, norepinephrine 30 min.
Baseline values were used as reference values (ref.) in the analysis to evaluate the time-based progression.
NE: Norepinephrine. LSPU: Laser speckle perfusion units. CI: Confidence Interval.
p-value for difference in LSPU compared at different timepoints to baseline, *indicates a p-value below 0.05


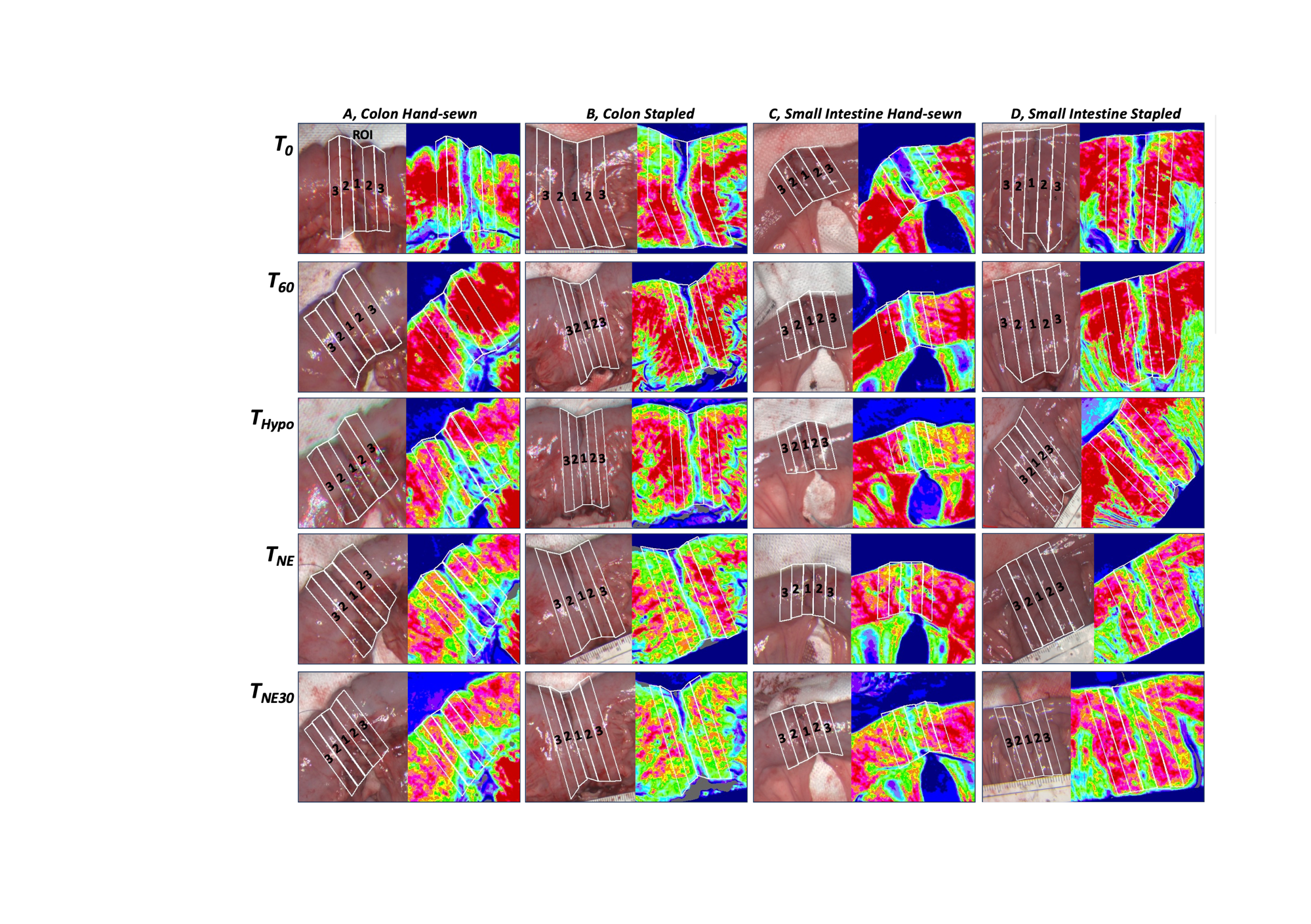
***SI Fig. 1. Examples of LSCI measurements of intestinal anastomoses: A, colon hand-sewn; B, colon stapled; C, small intestine hand-sewn; D, small intestine stapled.****The placement of regions of interest (ROIs) is indicated on all white light images using bold markings: ROIs were placed adjacent to each other, with ROI 1 centered on the anastomosis, ROI 2 ±5 mm on either side, and ROI 3 ±10 mm on either side of the anastomosis.
A color scale ranging from red to blue indicates good to poor perfusion, respectively.
T_B_=Baseline; T_0_=Immediately after anastomosis; T_60_= One hour with rest; T_Hypo_=Hypotension, MAP 50-60 mmHg; T_NE_=Restored MAP 85-100 mmHg with NE infusion; T_NE30_=After 30 minutes of MAP 85-100 mmHg with continuous NE.
MAP, mean arterial pressure; NE, norepinephrine.*
